# Blind spots in stigma research? Broadening our perspective on mental illness stigma by exploring ‘what matters most’ in modern Western societies

**DOI:** 10.1017/S2045796021000111

**Published:** 2021-03-17

**Authors:** G. Schomerus, M. C. Angermeyer

**Affiliations:** 1 Department of Psychiatry and Psychotherapy, University of Leipzig Medical Center, Leipzig, Germany; 2 Center for Public Mental Health, Gösing am Wagram, Austria

**Keywords:** values, mental illness stigma, cultural aspects, discrimination, social disadvantage

## Abstract

**Aims:**

The theory of ‘what matters most’ (WMM) has been developed to understand differences in mental illness stigma between cultures, postulating that stigma becomes most pervasive in situations that matter most in a specific cultural context. The rise of populism in Western societies demonstrates that also within one cultural context, different values ‘matter most’ to different groups. We expand the WMM framework to explore the spectrum of stigma manifestations within Western societies, relating it to both conservative/authoritarian and liberal/modern values. From our findings, we will develop hypotheses on how further research into value orientations and stigma might address potential blind spots in stigma research.

**Methods:**

Based on a narrative review of the literature on mental illness stigma and value orientations, we apply the WMM framework to cultural mechanisms of stigma within modern Western societies.

**Results:**

There are several studies showing an association between traditional, authoritarian, conservative values with stronger mental illness stigma, while studies examining the stigma within liberal, modern value orientations are scarce. We hypothesise on situations where encountering a person with mental illness could threaten liberal values and thus might provoke stigma among persons with such value orientations. For example, living with a person with mental illness could be seen as consuming energy and time, thereby jeopardising ‘self-actualisation’, the modern value of realising one's own full potential. As a result, a person highly valuing self-actualisation might try to avoid contact with persons with mental illness. Instances of potential ‘liberal stigma’ also include structural stigma or self-stigma, when, e.g. changing assumptions of what is considered ‘normal’ increase perceptions of being fundamentally different when experiencing mental illness.

**Conclusions:**

‘WMM’ appears to be a useful framework to direct research to potential blind spots within the field of stigma research. Looking at instances where liberal values conflict with dealing with a person with mental illness could provide a more comprehensive understanding of stigma experiences among persons with mental illness. However, for measuring stigma, tapping into liberal variations of mental illness stigma is methodologically challenging. Qualitative work could be the first step to elicit potential stigma experiences based on conflicts with liberal values.

## Introduction

### ‘What matters most’ (WMM) – understanding cultural differences in stigma

In their seminal article on ‘Culture and stigma: Adding moral experience to stigma theory’, Yang *et al.* (Yang *et al*., 2007) hypothesise that ‘Stigma … threatens the loss or diminution of what is most at stake’. Building on Kleinman's (2006) concept of ‘moral experience’, the authors emphasise the role of engagements in everyday social life that revolve around the preservation of fundamental values that ‘matter most’ to ordinary people. For remaining a fully recognised member of a local cultural group it is mandatory to participate in these activities. By diminishing the capacity to do this, stigma undermines a person's standing within the local group and, thus, endangers their personhood. ‘WMM, as Yang *et al.* later term their theory (Yang *et al*., 2014*b*), shapes both how stigma is experienced and the way stigma is enacted: For those who stigmatise, the necessity to use stigma to protect personal values or interactions might be greatest in situations that ‘matter most’. And for those stigmatised, stigma is particularly harmful by affecting the culturally most relevant interactions and situations. Yang *et al*. describe stigma as a ‘highly pragmatic, even tactical response to perceived threats, real dangers and fear of the unknown’ (Yang *et al*., 2007, p. 1528). ‘WMM’ gives a motive to stigma: people stigmatise persons with, for example mental illness in order to protect or advance those cultural values that are most important to them.

Stigma differs between cultures, and according to Yang *et al.*, these differences can be understood by identifying ‘WMM’ to persons within a shared cultural context, and contrasting it with ‘WMM’ in a different culture. So far, the ‘WMM’ theory has mostly informed research characterising stigma in non-Western cultures, often comparing it to Western culture or to stigma among persons of Western European descent. For example, a recent study characterising the stigma of human immunodeficiency virus among women in Botswana (Yang *et al*., 2021) identified themes related to being viewed as a ‘good woman’. Two representative population surveys compared the stigma towards a person with schizophrenia in Tunisia and Germany (Angermeyer *et al*., 2016), finding that the desire for social distance was greater in more distant relationships in Germany, while it was greater for close, family-related relationships in Tunisia, where family loyalty and obligations take precedence over loyalty to friends or demands of a job. Another study found ‘threat to family lineage’ is particularly relevant with regard to mental illness stigma among Chinese–Americans compared to European–Americans (Yang *et al*., 2013). A systematic review using the ‘WMM’ perspective to identify culturally salient aspects of mental illness stigma found ‘cultural ideals of the everyday activities that define personhood’ to be different between Asian groups (‘preserving ones lineage’), African–American groups (‘establishing trust among religious institutions due to institutional discrimination’) and Latino-American groups (‘fighting hard to overcome problems and taking advantage of immigration opportunities’). These essential cultural interactions were found to shape culturally salient stigma manifestations (Yang *et al*., 2014*a*).

### ‘WMM’ within a shared cultural context

Meaningful differences of ‘WMM’ to a group of persons, however, are not restricted to differences between cultures. Within Western societies, the rise of populism and an increasing polarisation of societies have demonstrated that quite different value orientations and cultural ideals are prevalent even within one cultural context. We argue that conceptualising these intra-cultural differences will help to broaden the scope of stigma research in a meaningful way. Applying the ‘WMM’ approach to groups that are distinguished by their values and ideals within the Western culture will probably enable identification of situations where stigmatisation of persons with mental illness occurs, that have so far not been fully acknowledged.

Several authors have pointed out that the apparent cleavages in Western societies are not primarily situated along a socio-economic axis between the rich and the poor. Instead, they see deepening cultural divisions, and a definition of ‘left’ and ‘right’ in cultural rather than economic terms. Norris and Inglehart, for example observe that traditional values of a once-dominant cultural majority have been threatened by a spread of cosmopolitan liberal orientations by growing minorities (Norris and Inglehart, 2019, p. 87–88). As a result, they see a deepening rift between authoritarian and libertarian cultural orientations. Goodhart (2017) contrasts ‘somewheres’, persons that are firmly connected to a specific community and are committed to traditional values, with ‘anywheres’, persons not being rooted in local communities, and entertaining liberal values, Merkel (2017) puts communitarians against cosmopolitans. These authors all refer to a cluster of traditional, national, authoritarian and culturally conservative values as opposed to modern, liberal, cosmopolitan values as defining a growing polarisation in Western societies. To capture this polarity of value orientations, we will refer to conservative or authoritarian *v*. liberal, cosmopolitan or modern values throughout this paper. This cultural division becomes even more visible as adherents of both world views tend to concentrate in different locations: While people endorsing modern values preferably live in big cities (where they tend to gravitate towards trendy districts), those endorsing traditional values prevail in small towns and rural hinterlands. Apart from this spatial segregation, and this seems to become ever more important, social media facilitate the creation of virtual communities which allow people to develop a sense of connectedness and where they can communicate with others who share the same values. As real communities, virtual communities can also function as ‘local worlds’ in which people are eager to maintain their social standing by enacting ‘WMM’.

### Aims of the study

We aim to determine whether different value orientations have been examined in relation to mental illness stigma. In particular, we are interested in whether stigma has been examined with regard to both liberal and conservative value orientations. Extending the ‘WMM’ theoretical framework to different value orientations within Western culture, we will develop hypotheses on how further research into value orientations and stigma might address potential blind spots of previous stigma research.

## Methods

For this conceptual article, we conducted a review of the literature related to value orientations and mental illness stigma, based on both databases (Pubmed, Web of Knowledge, Google Scholar) and bibliographies of identified articles. We used both general search terms like ‘value orientations’, and specific terms like conservative, liberal, authoritarian, progressive etc. Using an iterative approach, we further extended our search to specific values and situations that emerged as being conceptually relevant when applying ‘WMM’ to conservative and liberal value orientations. Since our aim is not to provide an exhaustive account of the literature related to values and mental illness stigma, but to expand an existing theoretical framework to identify areas that have potentially been understudied, our results will be presented as a narrative (rather than systematic) review.

## Results

Between the two opposing value orientations of conservative/authoritarian values and liberal/progressive values, the stigma of mental illness has primarily been examined with regard to its association with the former. Using scales like the right-wing authoritarianism (RWA) scale (Altemeyer, 1996) or the authoritarianism–conservatism–traditionalism (ACT) scale (Duckitt and Sibley, 2010), several studies found more conservative/authoritarian values to be related to mental illness stigma (Duckitt and Sibley, 2007; Kvaale and Haslam, 2016). Persons endorsing authoritarian values showed stronger endorsement of negative stereotypes (DeLuca and Yanos, 2016), and a preference for harsher punishment of a hypothetical offender with mental illness (Fodor *et al*., 2008). An experimental study found persons with higher RWA-scores having stronger negative attitudes towards a person with medically controlled schizophrenia at the workplace than persons with lower RWA-scores (Fodor, 2006). RWA has also been shown to be related to negative attitudes towards mental health services (Furr *et al*., 2003).

Closely related to value orientations are political attitudes (Rempala *et al*., 2016), with self-reported conservatism being associated with higher RWA scores (DeLuca *et al*., 2018), but also with adherence to tradition, resistance to change and justification of inequality (Jost *et al*., 2003). The social dominance orientation (SDO) scale (Pratto *et al*., 1994) has been developed to quantify a political preference for hierarchical relations between groups, putting one's own in-group in a superior position towards seemingly inferior out-groups (Pratto *et al*., 1994). SDO has repeatedly been shown to be associated with more stigma towards persons with mental illness (Duckitt and Sibley, 2007; Bizer *et al*., 2012; Kvaale and Haslam, 2016), for example with stronger dangerousness beliefs (Lampropoulos and Apostolidis, 2018) or a stronger desire for a social distance towards persons with schizophrenia (Phelan and Basow, 2007).

A study in Sweden demonstrated both conservative political ideology and support for conservative political parties to be associated with stronger stigmatising attitudes towards persons with depression (Löve *et al*., 2019). An online study in the United States found persons with liberal political ideology attributing less responsibility to a person with depression, showing more empathy and scoring lower on a depression stigma scale (Thibodeau *et al*., 2015). But not all studies found political orientation related to stigma: A study among the general public in Germany found right-wing extremism only marginally related to social distance towards persons with mental illness (Beck *et al*., 2005).

Only few studies have examined associations of stigma with a broader spectrum of different value orientations. Angermeyer and Matschinger examined a large representative sample of the German public and found respondents who endorsed more traditional values having a greater desire for social distance, while those endorsing more liberal (equality, social justice, tolerance) and more ‘modern’ (self-realisation, hedonism, post-materialism) values were more accepting of persons with mental illness (Angermeyer and Matschinger, 1997). Similarly, using the Schwartz Value Inventory (1992), Norman *et al.* found greater self-transcendence being associated with a lower desire for social distance from a person with mental illness, while self-enhancement or conservatism values were related to more social distance (Norman *et al*., 2008). Using the same value inventory, Skinner *et al*. (2007) found nurses with high self-transcendence values expressing more, and with high conservatism values expressing less positive affective responses towards patients with stigmatised conditions like drug-use. Traditional values have also been shown to be associated with more negative attitudes towards persons with alcohol use disorders, for example regarding resource allocation within health care services (Schomerus *et al*., 2006).

Previous studies thus paint a fairly consistent picture: traditional, authoritarian, conservative values are associated with more, and liberal/modern values are associated with less stigma. Some authors even propose a general prejudice factor, encompassing negative attitudes towards several minorities, being related to traditional, conservative, authoritarian values (Duckitt and Sibley, 2007).

### Traditional values and stigma

Looking at specific traditional values, the stigma literature is rich in findings suggestive of a close relationship with mental illness stigma. These inter-relations seem clearly shaped by ‘WMM’ for people holding these values. According to the motivated social cognition perspective, the endorsement of conservative values is in part determined by the way people perceive their social world (Jost *et al*., 2003). Persons who perceive the world to be threatening and dangerous are more inclined to espouse conservative ideologies (e.g. Van Leeuwen and Park, 2009, Shook et al., 2017). A view of the world as a dangerous place fosters the desire for security. Frequently endorsed stereotypes for schizophrenia include being dangerous and unpredictable (Pescosolido *et al*., 2019), and fear has been shown to be the emotion central to the stigma of schizophrenia (Angermeyer *et al*., 2010). The desire for security is thus threatened by these perceived attributes of someone with schizophrenia. Perceptions of dangerousness are accompanied by a strong perceived need for coercive treatment for this group of patients (Pescosolido *et al*., 2019). In fact, Watson *et al*. could show that conservatives are more likely to attribute bad character to a person with mental illness, which in turn was associated with perceptions of dangerousness and stronger support of legal coercion (Watson *et al*., 2005). Similar to fear, the stereotype of unpredictability (as well as that of lack of self-control) may also reflect a perceived threat to order and structure, which have been shown to be related to political conservatism, directly (Webster and Stewart, 1973) and indirectly through the endorsement of right-wing authoritarianism (Altemeyer, 1998; Peterson and Lane, 2001).

Another example is the belief that the world is a generally just place, where all people normally get what they deserve (Lerner, 1980), a belief which shows strong links to conservatism (Furnham and Procter, 1989). In consequence of the view that good things happen to good people and bad things to bad ones, sick people are at risk of being judged as responsible for their illness. In fact, *just world beliefs* tended to be associated with higher attributions of blame to persons with mental illness in a general population sample (Rüsch *et al*., 2010), supporting the hypothesis that stigma serves as a strategy to protect one's personal values: if people with mental illness are responsible for their illness, there is no need to question one's belief in a just world.

As a third example, values rooted in Protestant theology (Luther, Calvin) might increase mental illness stigma. *Protestant ethic*, a term coined by the German sociologist Max Weber (1904/1905), emphasises the importance of hard work and self-discipline for success in life. According to this doctrine, those who are successful deserve it because they have worked hard and are morally superior, whereas those who lack success deserve it because they are self-indulgent and morally flawed. Beliefs in Protestant ethic, which is related to higher levels of conservatism and authoritarianism (Feather, 1984), have been found to be associated with more perceived dangerousness, more negative stereotypes and more implicit guilt attributed to a person with mental illness (Rüsch *et al*., 2010).

Hence, it seems plausible that a conservative mindset, if it is rooted in values like those described, is associated with more stigma towards persons with mental illness.

### A blind spot in stigma research?

From the perspective of most academic scholars (including the authors), the finding that stigma seems to be a phenomenon associated with conservative values is reassuring, since it locates stigma within a group of ‘others’, those with traditional, conservative and authoritarian values, and not within the realm of liberalism, tolerance and cosmopolitanism (Inbar and Lammers, 2012). However, when taking a ‘WMM’ perspective, looking at persons with different value orientations within Western culture reveals a potential blind spot in previous, value related stigma research. If stigma impairs the most meaningful interactions in a certain cultural context, and is most apparent in situations that affect the individuals' most important values, the question arises whether stigma in situations and values relevant to persons with liberal values has been examined with similar rigour. We argue that there is a lack of stigma research capturing situations that are important to persons with liberal value orientations, and that hence potential discrimination of persons with mental illness in such situations might have been overlooked.

### Potential manifestations of stigma within a liberal cosmopolitan culture

The assumption that liberal values may foster stigma might, at first glance, appear counterintuitive. In fact, typical liberal values like inclusion of marginalised groups or valuing diversity are more likely to work against stigma than to encourage it. However, there are some liberal values which may have the opposite effect. In the following, we discuss some examples of how liberal values may intensify various forms of stigma (public stigma, structural stigma, self-stigma).

First, liberal values could decrease tolerance in situations that so far have not been evaluated as part of traditional stigma measures. A ‘modern’ value is striving for *self-actualisation* (Goldstein, 1940), or to realise one's own full potential. If living or dealing with someone with mental illness is perceived as an impediment to one's personal choices, or as consuming energy and time that would be needed to achieve self-actualisation, a person might try to avoid contact with persons with mental illness. Related, the value of *hedonism* might imply that ‘negative’ contacts should be avoided in order to increase one's personal happiness. People with mental illness could be regarded as a threat to one's personal happiness and individual wellbeing. As a result, people who highly value positivity might avoid closer contact with persons with mental illness. Or, if they have a relative or friend with mental illness, they might prefer professional mental health care over informal care within a family or group of friends.

Another example is *mobility*, which is a central value for ‘anywheres’, members of the urban middle class. Leaving one's parents' city and moving to new, attractive places in order to get an education and to take on new job opportunities is a central part of a successful cosmopolitan biography (Reckwitz, 2019, p. 92). Being close to someone with mental illness, however, might conflict with these personal goals. Presumably, persons who highly value mobility might endorse the stereotype of persons with mental illness being dependent on others, and fear that a close relationship to a person with mental illness might impede their own mobility. As a consequence, they could be more reluctant to engage in closer relationships with someone with mental illness.

There are other modern values or virtues like creativity, self-reliance, independence, all potentially being threatened by mental illness or by living with a person with mental illness, and potentially causing social withdrawal. The poet with lived experience Bill McKnight has concisely captured this motive for discrimination in his poem ‘Comfort zone’: ‘I don't want/To get involved with you – /You who are weak and upset. / Because you might upset me.’ (McKnight, 2012).

An area where a high emphasis on *achievement* or *self-actualisation* could foster separation is schooling. If providing one's children with optimal, competitive starting conditions or providing them with a space of undisturbed self-actualisation, are of great personal value, dealing with potentially difficult, challenging classmates with mental health problems threatens this value. In Germany, in spite of a generally well-functioning public school system, private schooling is on the rise, predominantly driven by school choices of middle-class parents who seek the best learning environment for their offspring (Koppetsch, 2019). Avoidance of classmates with difficulties due to their social backgrounds, or, often related, due to mental health problems could be a hidden motive here.

Second, liberal values could lead to structural discrimination of persons with mental illness. For instance, a consequence of the aforementioned trend towards segregation in schooling could be that young person with mental illness will be disadvantaged as regards access to higher education and their professional prospects. Another well-documented example is the not-in-my-backyard (NIMBY) syndrome (Wexler, 1996). Here, personal prestige and economic success are highly esteemed, so housing for persons with mental illness in one's neighbourhood is perceived as threatening the prestige and material value of one's property. A study in Germany found men with higher education being more strongly opposed to implementing community psychiatry services in their neighbourhood (Angermeyer and Matschinger, 1991). Of course, valuing a property is not restricted to persons with liberal values and does not even constitute a strictly liberal value. Rather, it seems to ‘matter more’ also to liberal residents than the tolerance that might on other occasions be stated as an important value to them. The NIMBY syndrome amounts to structural discrimination of persons in need of supported housing because they end up in less-privileged neighbourhoods with all their negative mental health consequences.

Third, modern values could increase both public stigma and self-stigma by changing assumptions of what is considered to be normal. If being flexible, productive, positively minded is the norm, being normal implies an even greater difference to having a mental illness. Baer *et al.* demonstrated in a qualitative discourse analysis using both interviews with people with mental illness and media reports, that the media carry an evolving norm of happy individuals characterised by being energised, motivated and powerful (Baer *et al*., 2016). This modern, individualistic norm puts persons with a mental illness like depression in even stronger opposition to being normal, thus increasing shame and self-stigma (Hahm *et al*., 2020; Rechenberg *et al*., 2020), but also increasing public notions of otherness, and potentially devaluation and discrimination. This would be an indirect, but powerful way of how modern liberal values could increase stigma.

## Conclusions

### Examining what matters most within Western culture and its relation to mental illness stigma

There is clear evidence that traditional, conservative values are associated with mental illness stigma in many forms, from negative stereotypes, stronger negative emotional reactions to stronger discrimination of persons with mental illness. However, the ‘WMM’ approach offers the opportunity to look for potential blind spots in stigma research, namely the question of whether there are instances where liberal values, too, could lead to stigmatisation of persons with mental illness. The challenge lies in measuring facets of the stigma associated with liberal values.

One way to look at ‘WMM’ with regard to mental illness stigma would be to create measures that tap on situations that are sensitive also to the fears of liberal respondents, where liberals may perceive their values to be in jeopardy through persons with mental illness. There are experimental studies in social psychology showing that liberal respondents, for example show levels of intolerance similar to conservative respondents, if both are asked about curtailing the rights of groups that are ideologically dissimilar to them (Brandt *et al*., 2014), like environmentalists to conservative people or anti-abortionists to liberal people. When intolerance towards persons with mental illness would be the focus of such studies, operationalising and varying situations where closer contact with a person with mental illness would threaten either liberal or conservative values would be necessary.

However, as we have discussed with regard to the NIMBY-syndrome, there might be particular difficulties with eliciting relevant attitudes from educated, liberal respondents, since attitudes stated in an interview might differ from attitudes and behaviour in real-world situations. Using the ‘WMM’ perspective, one could even argue that in an interview situation, self-affirmation as being tolerant and liberal could be what matters most – while in real-life situations other values like advancing the prospects of one's children could be more important. Competing values might also simultaneously influence how we act in certain situations: the desire to achieve self-actualisation might compete with the desire to support a close person with mental illness, leading to mixed outcomes like, for example ‘reluctant help’ or other subtle forms of stigma that are difficult to measure, but nevertheless consequential for someone with mental illness. Measuring liberal variations of mental illness stigma thus remains a challenge. Implicit measures as well as experimental designs allowing for gradual behavioural responses could yield novel insights. But probably, starting with qualitative research eliciting when and how people experience being stigmatised in a context determined by liberal value orientations should be a first step that may provide a way to more fully understand the impact of stigma on persons with mental illness in western societies.

### Implications for anti-stigma interventions

Most anti-stigma campaigns related to mental illness stigma have so far addressed the general public, or specific stakeholders, but have not differentiated between political or culturally defined subgroups of the population. However, as we have argued in this paper, in polarising societies, where a cultural divide between groups committed to traditional values seems from groups favouring liberal values is growing, these values define ‘WMM’ in the cultural context where they are most salient. If core lived values appear threatened through people with mental illness, stigma results (Yang *et al*., 2007). Anti-stigma interventions should therefore focus on what is fundamentally at stake in a given local setting. Rather than being prescribed by psychiatric experts, who run the risk of turning a blind eye to potential negative effects of their own (often liberal) values, interventions should be based on the local stigma experiences of people with mental illness – quite in line with Corrigan's demand that stigma needs to be changed locally, both in a geographical and cultural sense (Corrigan, 2011). Applying ‘WMM’ might improve our understanding and conceptualisation of stigma experiences in culturally defined subgroups, and it might clarify which situations need to be addressed in order to empower people with mental illness to recover their personhood.

## Data Availability

Not applicable.
